# Elevating Educational Research: A Focus on Objectives, Operations, and Outcomes Model

**DOI:** 10.7759/cureus.56937

**Published:** 2024-03-26

**Authors:** Mohammad I Qureshi, Tripti Shrivastava, Vedprakash Mishra, Gaurav V Mishra

**Affiliations:** 1 Neurophysiotherapy, Ravi Nair Physiotherapy College, Datta Meghe Institute of Higher Education & Research, Wardha, IND; 2 Physiology, Datta Meghe Institute of Higher Education & Research, Wardha, IND; 3 Radiodiagnosis, Datta Meghe Institute of Higher Education & Research, Wardha, IND

**Keywords:** evidence based practice, medical education, quest, triple o model, research framerwork, educational research

## Abstract

The Triple “O” model in educational research, meaning the Objectives, Operations, and Outcomes model, is a plan to dive into how research works. Objectives are the reasons for this research, directing what researchers aim to explore. Operations focus on transforming these reasons into actual steps; they navigate the methods for gathering information. As for the Outcomes, they represent significant shifts resulting from the research, such as improved methods of teaching or students achieving their educational targets with better learning outcomes. Using the Triple “O” model, researchers are expected to explore educational research and studies significantly and effectively. This model provides a clear plan on how to conduct research and pay attention to the results. It is ready to change the whole scene of educational research and make good, important differences in learning.

## Editorial

Research is a methodical and structured process of inquiry that seeks to answer a variety of problems, provide new information, and enhance the current understanding. It is methodological research that entails collecting data, analyzing and interpreting certain questions, or advancing a subject of study. Information gathering for research must be methodological and planned. It employs a methodological approach that includes defining the research problem, examining pertinent literature, developing research questions or hypotheses, gathering and evaluating data, and arriving at conclusions. Instead of depending on conjectures, research is based on empirical evidence, observations, and data. Empirical evidence supports the findings of this study and enables study replication to confirm the findings [1].

A subfield of research known as “educational research” is a systematic investigation into educational issues, practices, policies, or phenomena with the aim of improving understanding and informing decision-making in the field of education. It covers a broad range of subjects, including instructional strategies, curriculum development, learning objectives for students, educational policy, and the influence of socioeconomic variables on education. By offering evidence-based insights that support policy creation and decision-making processes in educational institutions, educational research aims to improve the quality of education [2].

Educational research has a big impact on education now and in the future. Numerous stakeholders, including parents, lawmakers, educators, and children, have gained from it. There are some primary arguments in favor of educational research’s significance: empirical data from educational research supports educators in making well-informed decisions. For instance, a study might investigate the effectiveness of different teaching methods, and the empirical data collection and analysis can provide evidence-based recommendations for a strategy. Educators, administrators, and lawmakers use research findings to support their decisions on curriculum development, instructional practices, and educational policies [3].

It is important to shift from opinion-based research to evidence-based research, which was implemented more than a couple of decades ago. Best Evidence Medical Education (BEME) involves the use of approaches and methods for education in such a way that it determines the best available evidence. This involves the determination of the Quality, Utility, Intent, Strength, Target, and Setting (QUEST) dimensions, which are implemented according to the educator’s professional experience. The dimensions highlight quality in terms of reliability and utility in terms of validity and are adopted without any alterations. The model also encompasses its applicability and relevance in educational research [4].

Conceptual models and theories provide a structure for future research. Research without a theoretical base provides isolated information that cannot be effectively used or applied. A research framework guides researchers in developing research questions, refining their hypotheses, selecting interventions, and defining and measuring variables [5]. Developing a research framework is a constantly evolving process that will always lead to a paradigm shift in evidence-based practice.

Why this model

The Triple “O” model, which is the Objectives, Operations, and Outcomes model, is a conceptual framework that captures the core of educational research through its three key components: Objectives, Operations, and Outcomes. This model guides researchers from setting research goals to achieving meaningful educational outcomes.

Objectives

The purpose of our model is to crystallize its purpose. Meticulously defined and sharply focused objectives serve as guiding stars that steer the trajectory of educational research. They provide compass points, directing inquiries, investigations, and interventions toward the realms of utmost significance within the educational landscape.

Operations

Connecting the conceptual and applied components, the operations component translates objectives into actionable strategies. This component focuses on the “how” of research, encapsulating methodologies, data collection, and the intricacies of implementation. Operations are gears that set the machinery of the research in motion, ensuring the alignment of actions with the overarching objectives.

Outcomes

The culmination of objectives and operations materializes in the realm of outcomes. This facet encapsulates the transformative impact of research, ranging from enhanced teaching practices to improved student learning outcomes. Therefore, outcomes become the litmus test for the efficacy of educational research, embodying the tangible manifestations of scholarly endeavors.

As we conclude in the development phase of the Triple “O” model for educational research, the future holds promising opportunities for transformative impact in various domains of education, e.g., curriculum, teaching and learning methods, assessment, evaluation, skills, and other competencies. The model, designed to address gaps in the research methodology and outcome focus, is poised to shape the future landscape of educational research. Envisioning as a catalyst for positive change and its adoption and integration into research practices is expected to enhance the quality and relevance of research outcomes.
